# Outcome of Thrombotic Thrombocytopenic Purpura Patients: A Single-Center Experience

**DOI:** 10.4274/tjh.galenos.2019.2019.0048

**Published:** 2019-08-02

**Authors:** Özcan Çeneli, Seda Yılmaz, Mehmet Ali Karaselek, Kazım Çamlı

**Affiliations:** 1 Necmettin Erbakan University, Meram Faculty of Medicine, Department of Hematology, Konya, Turkey

**Keywords:** Thrombotic thrombocytopenic purpura, Plasma exchange, ADAMTS13, Rituximab

## To the Editor,

Thrombotic thrombocytopenic purpura (TTP) is a rare, life-threatening condition [1,2]. It is characterized by platelet-rich thrombi in the microcirculation caused by severely decreased activity of the von Willebrand factor-cleaving protease ADAMTS13 (a disintegrin and metalloprotease with thrombospondin type motif 13), leading to the accumulation of ultra-large von Willebrand factor multimers, microangiopathic hemolytic anemia, and sometimes organ damage. TTP can be acquired due to autoantibody inhibitor development against *ADAMTS13*, or it can be hereditary due to inherited mutations in *ADAMTS13*. Hereditary TTP represents less than 5% of all TTP cases; over 95% are cases of acquired autoimmune TTP [3]. TTP is a hematologic emergency that is almost always fatal if appropriate treatment is not initiated promptly, and even with treatment, the mortality can reach 10% to 20% [1].

In our retrospective study we aimed to investigate the factors affecting the outcome of TTP patients. Written informed consent was obtained from all patients. Nineteen TTP patients (11 females and 8 males) had a mean age of 41.5±12.7 (18-60) years; 12 (63.1%) had neurologic features, 4 (21.1%) fever, and 3 (15.7%) renal impairment (Table 1). All patients received plasma exchange (PEX) therapy within 5 h of admission. Eighteen (94.7%) patients received 1 mg/kg adjunctive methylprednisolone (except for one hereditary TTP patient). One refractory patient and two relapsed patients received rituximab. Statistical analyses were performed with Jamovi 0.9.2.6 software. We used Kruskal-Wallis and Mann-Whitney U tests to examine the mean differences. A p-value of <0.05 was considered statistically significant.

Laboratory results are presented in Table 2. Relapsed/refractory patients and non-relapsed/refractory patients were compared in terms of number of PEX sessions until obtaining remission, laboratory values, and ADAMTS13 panel.

In conclusion, three interesting results were identified after analysis of data in our study. First, our overall mortality rate was 1 in 19 (5.3%). Higher mortality rates were reported in previous studies (10%-20%) [1,2]. This result may show that early PEX initiation is an effective factor in mortality reduction. Secondly, the mean d-dimer value of our TTP patients was higher than the reference limit at 2.65 µg/mL (reference values: 0-0.4 µg/mL). Thus, in cases of slightly elevated d-dimer levels, one should not hesitate to start urgent PEX treatment in patients with clinically high suspicion of TTP if *ADAMTS13* panel results are not obtained quickly. Thirdly, relapsed/refractory patients needed more PEX sessions to achieve first remission. A smaller number of PEX sessions to achieve response may be predictive of durable remission without relapse.

## Figures and Tables

**Table 1:**
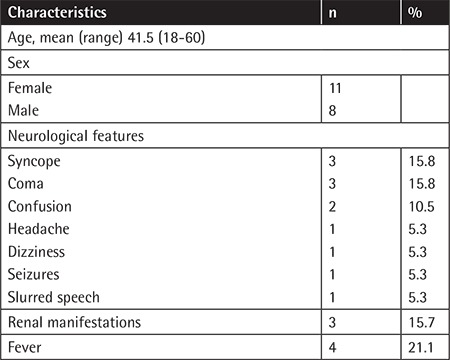
Characteristics of the patients.

**Table 2:**
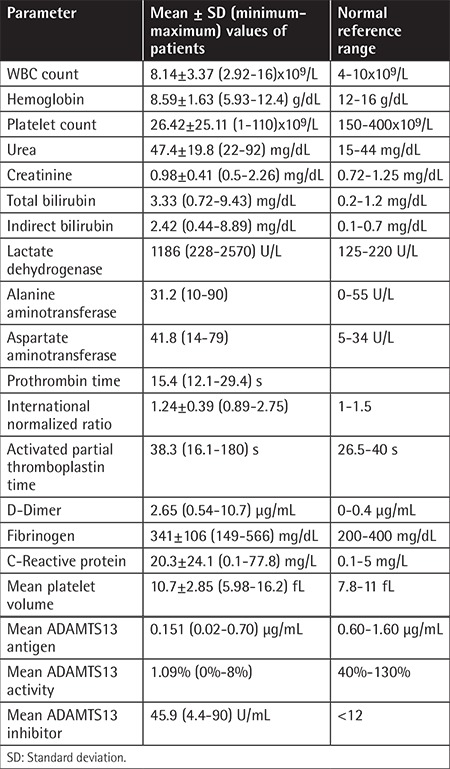
Laboratory results.
